# [^11^C]MADAM Used as a Model for Understanding the Radiometabolism of Diphenyl Sulfide Radioligands for Positron Emission Tomography (PET)

**DOI:** 10.1371/journal.pone.0137160

**Published:** 2015-09-14

**Authors:** Fabienne Gourand, Nahid Amini, Zhisheng Jia, Sharon Stone-Elander, Denis Guilloteau, Louisa Barré, Christer Halldin

**Affiliations:** 1 Karolinska Institutet, Department of Clinical Neuroscience, Centre for Psychiatric Research, SE-171 76 Stockholm, Sweden; 2 CEA, DSV/I2BM, LDM-TEP Group, GIP Cyceron, Bd Henri Becquerel, BP 5229, F-14074 Caen, France; 3 Université de Caen Basse-Normandie, Caen, France; 4 CNRS, UMR ISTCT 6301, LDM-TEP Group, GIP Cyceron, Caen, France; 5 Neuroradiology, Karolinska University Hospital, MicroPET and Clinical Neurosciences, Karolinska Institutet SE-171 76 Stockholm, Sweden; 6 INSERM U930- Université François Rabelais de Tours, CHRU de Tours, 2 boulevard Tonnellé, 37044 Tours, France; Wayne State University, UNITED STATES

## Abstract

In quantitative PET measurements, the analysis of radiometabolites in plasma is essential for determining the exact arterial input function. Diphenyl sulfide compounds are promising PET and SPECT radioligands for *in vivo* quantification of the serotonin transporter (SERT) and it is therefore important to investigate their radiometabolism. We have chosen to explore the radiometabolic profile of [^11^C]MADAM, one of these radioligands widely used for *in vivo* PET-SERT studies. The metabolism of [^11^C]MADAM/MADAM was investigated using rat and human liver microsomes (RLM and HLM) in combination with radio-HPLC or UHPLC/Q-ToF-MS for their identification. The effect of carrier on the radiometabolic rate of the radioligand [^11^C]MADAM *in vitro* and *in vivo* was examined by radio-HPLC. RLM and HLM incubations were carried out at two different carrier concentrations of 1 and 10 μM. Urine samples after perfusion of [^11^C]MADAM/MADAM in rats were also analysed by radio-HPLC. Analysis by UHPLC/Q-ToF-MS identified the metabolites produced *in vitro* to be results of *N*-demethylation, *S*-oxidation and benzylic hydroxylation. The presence of carrier greatly affected the radiometabolism rate of [^11^C]MADAM in both RLM/HLM experiments and *in vivo* rat studies. The good concordance between the results predicted by RLM and HLM experiments and the *in vivo* data obtained in rat studies indicate that the kinetics of the radiometabolism of the radioligand [^11^C]MADAM is dose-dependent. This issue needs to be addressed when the diarylsulfide class of compounds are used in PET quantifications of SERT.

## Introduction

**Fig 1:**
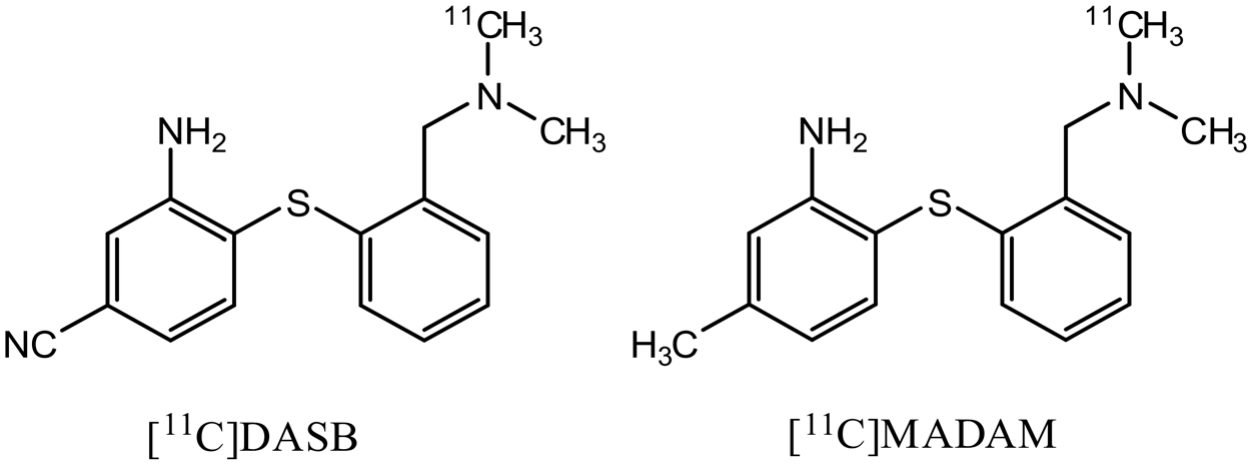
Chemical structures of [^11^C]DASB and [^11^C]MADAM.

The serotonin transporter (SERT, also known as 5-HTT) has been implicated in a variety of neurological and psychiatric disorders such as depression, schizophrenia, mental illness and neurodegenerative pathologies such as Parkinson’s and Alzheimer’s diseases. *In vivo* positron emission tomography (PET) imaging of the SERT is a valuable tool in monitoring diseases in which serotoninergic function is altered. A number of SERT radioligands have been developed [1–7]. Ligands with a core diaryl sulfide structure have high SERT-binding affinity, notable brain uptake and selective regional brain localization and yield promising results in *in vivo* PET studies. Amongst them, [^11^C]*N*,*N*-dimethyl-2-(2-amino-4-cyanophenylthio)benzylamine ([^11^C]DASB) and [^11^C]*N*,*N*-dimethyl-2-(2-amino-4-fluoromethylhenylthio)benzylamine ([^11^C]MADAM) (Fig 1) are putative radioligands for investigations of the complex SERT in patients [5–7].

Radiometabolite analyses of human and non-human plasma samples by radio-HPLC have previously shown rapid peripheral metabolism of [^11^C]MADAM [6, 7]. The percentage of the unchanged radioligand [^11^C]MADAM in human plasma was approximately 40%, 50 min after injection of the radioligand [7]. Since diphenyl sulfide compounds are potentially the most promising candidates for the detailed quantification of the SERT, their radiometabolism needs to be further understood. Variations in the radiometabolism of a radioligand will affect the concentration of the radioligand in plasma, which has importance for the quantification in PET studies. To our knowledge, one PET study performed in humans has highlighted different rates of radiometabolism of a radioligand depending on the administered dose: the percentage unchanged [^11^C]SCH 39166 (used for visualization of dopamine-D1 receptor) in plasma at 60 minutes after injection, was approximately 30% and 15% at high and low specific radioactivity, respectively [8]. These results suggest a dose-dependency regarding the radiometabolism of this radioligand. In the present study, our efforts focused on investigating the radiometabolic pathways of [^11^C]MADAM and structural elucidation of its radiometabolites. Metabolite identification in *in vivo* PET studies is challenging since the amount of compound administered is in the subnanomolar range. Thus identification of the radiometabolites in plasma is a complex task. Drug metabolism occurs mainly in the liver and radiopharmaceuticals, similar to other drugs, undergo phase I and II metabolism. *In vitro* assays are convenient for studying drug metabolism. In particular, liver microsomes have been routinely used for examining drug biotransformation. In addition, liquid chromatography coupled with mass spectrometry is a powerful analytical tool for screening and identifying drug metabolites in biological matrices. Accordingly, to analyze the *in vitro* metabolism of the radioligand [^11^C]MADAM, we used human and rat liver microsomes (HLM and RLM) combined with UHPLC/Q-ToF-MS for their identification [9]. Although this method is a promising approach to understand the radiometabolism of MADAM, it may not entirely reflect the complexity of the *in vivo* situation. Therefore, *in vivo* studies in rats were also undertaken to further study the dose-dependency of the radiometabolism of the radioligand which was revealed in the RLM and HLM experiments.

## Materials and Methods

### Chemicals and reagents

HLM and RLM containing 20 mg protein/mL and nicotinamide adenine dinucleotide phosphate (NADPH) were purchased from Sigma-Aldrich. Water and acetonitrile (both LC-MS grade) were obtained from Fisher Scientific, while the other solvents used were from Aldrich. The compounds MADAM, NHMADAM, SOMADAM, NHSOMADAM and SO_2_MADAM were obtained as reported previously [10]. [^11^C]MADAM was synthesized from [^11^C]methyl triflate ([^11^C]CH_3_OTf) [6].

### HLM and RLM incubation procedure

MADAM and/or [^11^C]MADAM (5–15 MBq) were incubated with HLM and RLM (0.5 mg/mL) at 37°C in 1 mL of 0.05 M potassium phosphate buffer (pH 7.4) containing 5 mM NADPH. Experiments were performed with MADAM concentrations of 1 and 10 μM. The incubation times were the following: 1 and 4 min (RLM experiments); 10, 45 and 90 min (HLM experiments). The incubation was stopped by adding an equal volume of ice-cold acetonitrile. The mixture was then vortexed and centrifuged and the supernatant was removed, evaporated. The residue was reconstituted in 150 μL mobile phase before being analyzed by radio-HPLC and UHPLC/Q-ToF-MS as described below. Control incubations in the absence of microsomes, MADAM or NADPH were also performed to assure that the observed peaks corresponded to MADAM metabolites.

### Radio-HPLC conditions

Reversed phase HPLC was used to determine the percentages of radioactivity in microsomal incubation samples that corresponded to unchanged radioligand and its radiometabolites. The HPLC system consists of a Merck Hitachi D-7000 pump, an interface module, a Merck Hitachi L-7400 UV absorbance detector (254 nm) in series with a Packard Radiomatic 150TR radiodetector equipped with a 600 μL flow cell, and a Rheodyne 7125 manual injector. A Waters μ-Bondapak C18 column (7.8 x 300 mm, 10 μm) with acetonitrile (A) and ammonium formate 0.1M (B) as the mobile phase was used with a flow rate of 6.0 mL/min, according to the following gradient: 0–4.5 min, (A/B) 10/90-15/85; 4.5–5.0 min, (A/B) 15/85-20/80; 5.0–5.5 min (A/B) 20/80-45/55; 5.5–9.0 min (A/B) 45/55; 9.0–10 min (A/B) 45/55-70/30; 10.0–20.0 min (A/B) 70/30. The detected peaks were integrated and their areas were expressed as a percentage of the sum of areas of all radioactive compounds present (decay-corrected).

### UHPLC/Q-ToF-MS conditions

The analyses were performed on a Waters (Milford, MA, USA) Acquity Ultra Performance LC^TM^ binary solvent manager coupled to a photodiode array detector and Waters (Micromass UK Limited, Manchester, UK) Q-Tof Premier. All the samples (10 μL) were injected onto a Waters Ethylene Bridged Hybrid (BEH) C18 column (2.1 x 50 mm, 1.7 μm) and eluted by a 5 min linear gradient starting from 100% water containing 0.1% formic acid and ending with 30% acetonitrile containing 0.1% formic acid at a flow rate of 0.5 mL/min. Positive electrospray ionization (+ESI) in V-mode with an extended dynamic range was used under the following conditions: capillary 3.5 kV, sampling cone 25 V, extraction cone 4.5 V, source temperature 100°C and desolvation temperature 380°C. Two scan functions, MS and MS^E^, in the mass range of 100–1000 Da, were performed simultaneously. The collision energy was set to 5 eV during the MS acquisition and it was ramped from 10 to 35 eV during the MS^E^ acquisition. MetaboLynx^TM^ (Waters, Milford, MA, USA) was used to aid metabolite identification.

### In vivo studies in the rat

All animal handling and experiments were carried out in accordance with the guidelines of Karolinska Institutet and were approved by the local laboratory animal ethics committee (N 363/05 and N 373/07). The rats were housed under standard laboratory conditions with free access to laboratory food and water *ad libitum*. Male Sprague-Dawley rats were anesthetized with isoflurane, via an E-Z anesthesia vaporizer (5% initially and then 1.5% to maintain anesthesia, blended with 7:3 air: O_2_ and delivered through a Microflex non-rebreathing mask from Euthanex Corporation, Palmer, PA. The rats were placed on a heating pad (37°C) while [^11^C]MADAM (52–76 MBq) and/or MADAM (25 μg to 1 mg) as a perfusion were administered intravenously; the rats were sacrificed at various time points after the administration (15, 30 and 60 min). Urine samples were collected at each time point and acetonitrile (400 μL) was added. After centrifugation at 3000g for 4 min, the supernatant was injected into the radio-HPLC (section 2.3). The radioactivity of the precipitate was measured to quantify the efficiency of the acetonitrile extraction.

## Results and Discussion

### In vitro RLM and HLM incubations

In this study, labeled metabolites of MADAM and/or [^11^C]MADAM in RLM and HLM were distinguished by on-line HPLC-radioactivity detection. Initially, no-carrier-added [^11^C]MADAM was incubated with RLM and HLM in the presence of NADPH and the percentage of unchanged [^11^C]MADAM was determined at designated times (1 and 4 min in RLM experiments; 10, 45 and 90 min in HLM experiments). No radiometabolites were detected when control incubations were performed without either NADPH or microsomes, indicating that cytochrome P-450 is accountable for the biotransformation.

The radiometabolism of [^11^C]MADAM in the presence of RLM was rapid; only 3.3 ± 1% of [^11^C]MADAM was left after 1 min (Table 1) and no [^11^C]MADAM was detected after an incubation time of 4 min. The same procedure was repeated with HLM, in which the percentage of intact [^11^C]MADAM decreased from 51 ± 4% at 10 min to 35 ± 1% at 45 min and to 16 ± 1% at 90 min (Table 1). Our results are comparable with the data reported from *in vivo* human PET studies [7], where approximately 40% intact [^11^C]MADAM was present in plasma 50 min after administration.

**Table 1:** Percentages of unchanged [^11^C]MADAM at various time points after incubation with either rat (RLM) or human (HLM) liver microsomes.

Incubation Time (min)	1	4	10	45	90
**[** ^**11**^ **C]MADAM (%) (RLM)**	3.3 ± 1%^1^	n.d.^2^	n.a.	n.a.	n.a.
**[** ^**11**^ **C]MADAM (%)(HLM)**	n.a.^3^	n.a.^3^	51 ± 4%	35 ± 1%	16 ± 1%

^1^ Values reported are (mean ± standard deviation) of three measurements.

^2^ n.d. not detected.

^3^ n.a. not analysed.

To characterize the formed metabolites using UHPLC/Q-ToF-MS, addition of 10 μM carrier to the incubation medium was essential to reach the limit of detection (LOD) of the instrument. In the presence of carrier (10 μM), the radiometabolism of [^11^C]MADAM decreased drastically and 78 ± 4% intact [^11^C]MADAM was present after 1 min of incubation with RLM (in comparison to 3.3 ± 1% with no-carrier-added) and 80 ± 5% (in comparison to 35 ± 1% with no-carrier-added) after 45 min of incubation with HLM (Table 2).

**Table 2:** Percentages of unchanged [^11^C]MADAM after incubation with rat (RLM) or human (HLM) liver microsomes in the presence of carrier at two different concentrations.

	Intact [^11^C]MADAM (%)	
	MADAM (1 μM)	MADAM (10 μM)
**RLM (t = 1 min)**	10 ± 2%^1^	78 ± 4%
**HLM (t = 45 min)**	58 ± 2%	80 ± 5%

^1^ Values reported are (mean ± standard deviation) of three measurements.

t = Incubation time.

To examine the effect of carrier on the radiometabolic rate of [^11^C]MADAM in more detail, incubations were carried out with a lower carrier added concentration of 1 μM and the findings were compared to those with 10 μM (Table 2). Under these conditions, the percentage of intact [^11^C]MADAM was 10 ± 2% after 1 min of incubation with RLM whereas this percentage was 58 ± 2% after 45 min of incubation with HLM. These results suggest that the *in vitro* radiometabolism of [^11^C]MADAM is dose-dependent. Indeed, significant changes in the radiometabolism rate of the radioligand [^11^C]MADAM were observed in RLM and HLM experiments under various conditions: a) incubation with the no-carrier-added radioligand [^11^C]MADAM alone; b) incubation with [^11^C]MADAM and MADAM (1 μM); and c) incubation with [^11^C]MADAM and MADAM (10 μM).

### Identification of MADAM metabolites by UHPLC/Q-ToF-MS

**Fig 2:**
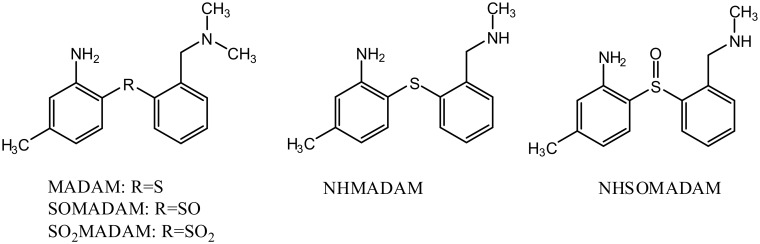
Chemical structures of MADAM, SOMADAM, SO_2_MADAM, NHMADAM and NHSOMADAM.

**Fig 3:**
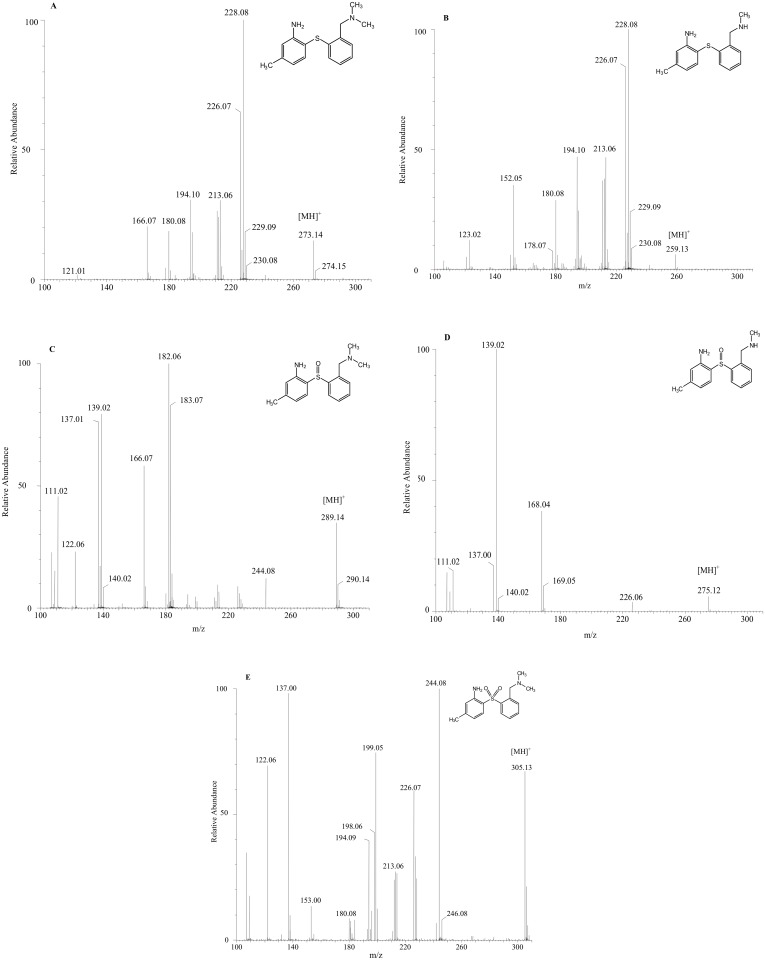
MS^E^ spectra and structures of the synthesized reference compounds. (A) MADAM, (B) NHMADAM, (C) SOMADAM, (D) NHSOMADAM and (E) SO_2_MADAM.

Diphenyl sulfide compounds have been shown to be oxidized by cytochrome P450 oxidoreductase and/or by flavin-containing monooxygenases into their corresponding sulfoxide derivatives and thereafter into their sulfone derivatives [11–12]. Consequently, it could be hypothesized that MADAM would first oxidize to SOMADAM and then further to SO_2_MADAM (Fig 2). These potential metabolites, SOMADAM and SO_2_MADAM, were synthesized in a previous study [10]. Two other potential metabolites, NHMADAM and NHSOMADAM (Fig 2), are available as reference compounds and have been used as precursors for the carbon-11 labelling of PET radioligands [^11^C]MADAM and [^11^C]SOMADAM [10]. To identify the metabolites of MADAM produced *in vitro*, the available synthesized references were analyzed by UHPLC/Q-ToF-MS in MS and MS^E^ modes simultaneously and their chemical structures and MS^E^ spectra are presented in Fig 3.

As mentioned previously, the rate of metabolism of MADAM is markedly influenced by its concentration. However, to identify the metabolites of MADAM produced *in vitro* it was necessary to use a relatively high concentration of MADAM (10 μM). The metabolites formed after 30 and 60 min incubation of MADAM with RLM and HLM respectively, were identified by comparing their retention times and fragmentations to the available synthesized reference compounds, Table 3.

**Table 3:** List of metabolites identified after incubation of MADAM with RLM (incubation time: 30 min) and HLM (incubation time: 60 min). The retention time, m/z of parent and major fragment ions for each compound are listed.

Compound Abbreviation	Retention time (min)	Molecular ion (m/z)	Major fragment ions (m/z)
**MADAM**	4.3	273.14	228.08, 213.06, 194.10, 166.07
**NHMADAM**	4.2	259.13	228.08, 213.06, 194.10, 180.08, 152.05
**SOMADAM**	3.0	289.14	182.06, 166.07, 139.02, 111.02
**NHSOMADAM**	2.9	275.12	168.04, 139.02, 111.02
**Hydroxyl-MADAM**	2.5	289.14	271.14, 244.06, 226.06, 212.04
**Hydroxyl-NHMADAM**	2.2	275.12	257.12, 244.07, 227.05, 210.09, 184.03

**Fig 4:**
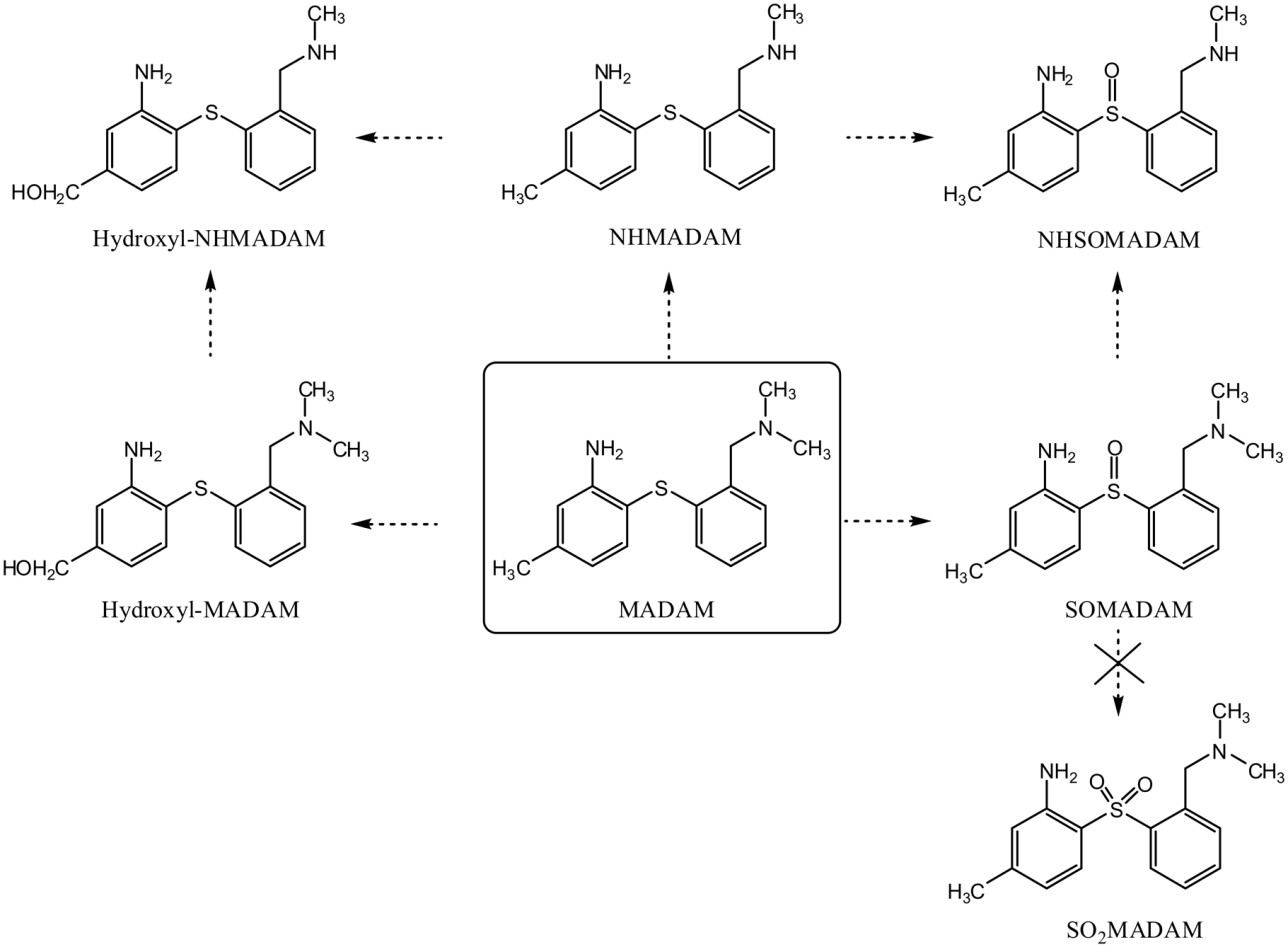
Proposed *in vitro* metabolic pathways of MADAM.

Incubation with either HLM or RLM resulted in five major metabolites, of which three were identified using the synthesized reference materials and structures were proposed for the other two by means of their fragmentation patterns, Fig 4. *N*-Demethylation (NHMADAM; m/z 259.13) and *S*-oxidation (SOMADAM; m/z 289.14), individually or combined (NHSOMADAM; m/z 275.12), resulted in the three more hydrophobic metabolites which corresponded to the available references. The other two metabolites, for which no references were available, were formed by the oxidation of the benzylic methyl group of MADAM and NHMADAM leading to hydroxyl-MADAM (m/z 289.14) and hydroxyl-NHMADAM (m/z 275.12) respectively. The hydroxylation of benzylic compounds by hepatic microsomes is to be expected and has been reported repeatedly [13]. The fragment with the m/z of 271.14, formed by the loss of H_2_O, is characteristic of a benzylic hydroxylation and thus suggests that the hydroxyl group is not on the aromatic ring. As shown in Table 3, the mass spectral fragmentation of MADAM (m/z 273.14) generated a major fragment at m/z 228.08 resulting from the loss of NH(CH_3_)_2_. An increase of 16 Da of these two product ions led to m/z 289.14 and 244.06, which are observed in the compound hydroxyl-MADAM (Table 3).

In the metabolite hydroxyl-NHMADAM (m/z 275.12), a similar fragmentation pattern was observed, where fragments m/z 257.12 and m/z 244.07 lose H_2_O and NH_2_CH_3_ respectively. SO_2_MADAM (Fig 4) was not detected in any of the microsomal preparations. In previous PET studies, a minor *in vivo* peripheral oxidation of [^11^C]MADAM to [^11^C]SOMADAM was noted in macaque plasma [10]. This fraction corresponded to 5% of the total radioactivity 4 min after injection and 1% after 15 min. No further oxidation to [^11^C]SO_2_MADAM was detected. By comparing our present results to a previous publication [9], it becomes apparent that the metabolic behaviour of diphenyl sulfides *in vivo* can be successfully predicted from their *in vitro* metabolism by hepatic microsomes.

**Fig 5:**
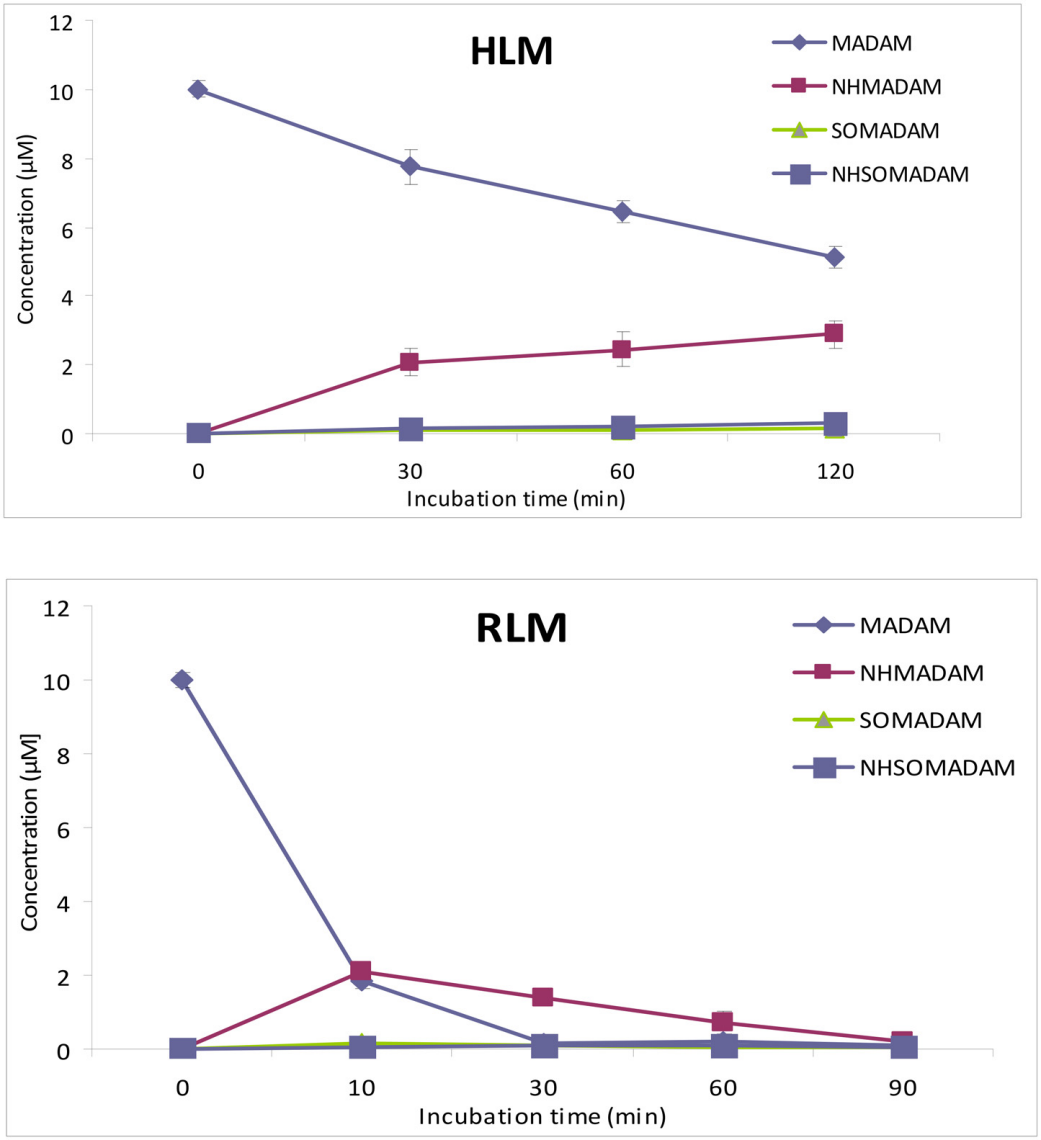
Concentrations of MADAM, NHMADAM, SOMADAM, NHSOMADAM produced by HLM and RLM at various incubation times.

In the present study, we further aimed to estimate the concentration of the ligand (MADAM) as well as that of the identified metabolites, among them NHMADAM, SOMADAM and NHSOMADAM, for which reference compounds were available. UHPLC/Q-ToF-MS instrumentation was employed to quantify these compounds in RLM and HLM incubations using AFM as the internal standard. In HLM incubations, the concentration of MADAM decreased from 10 μM to 7.76 ± 0.5 μM at 30 min and approximately half of MADAM (5.1 ± 0.5 μM) was still present after an incubation time of 120 min. As shown in Fig 5, the demethylated product NHMADAM was the major metabolite observed and the concentration of the latter increased from 2.08 ± 0.40 μM to 2.89 ± 0.40 μM at incubation times of 30 and 120 min, respectively. Negligible amounts of the other two metabolites, SOMADAM and NHSOMADAM, were detected at these times. The concentration of SOMADAM ranged from 0.12 ± 0.02 μM to 0.15 ± 0.01 μM over time. A very slight increase in the concentration of NHSOMADAM was observed, from 0.12 ± 0.01 μM to 0.32 ± 0.03 μM at 30 min and 120 min incubation times, respectively.

Concentrations of MADAM, NHMADAM, SOMADAM and NHSOMADAM were determined at different incubation times in the RLM experiments, as displayed in Fig 5. The parent compound MADAM was present at a concentration of 1.83 ± 0.20 μM at 10 min and from 30 min the concentration was very low (0.17 to 0.09 μM), indicating a fast metabolism in RLM. Interestingly, the profile for metabolites NHMADAM, SOMADAM and NHSOMADAM observed in RLM incubations was similar with that of HLM experiments: the two metabolites SOMADAM and NHSOMADAM were found at very low concentrations and the metabolite NHMADAM was the major metabolite detected. However, the concentration of the latter declined to 2.08 ± 0.3 μM to at 10 min and to 0.72 ± 0.31 at 60 min. The quantification in HLM experiments was not undertaken after 120 min.

### In vivo dose-dependency of radiometabolism of [^11^C]MADAM

**Fig 6:**
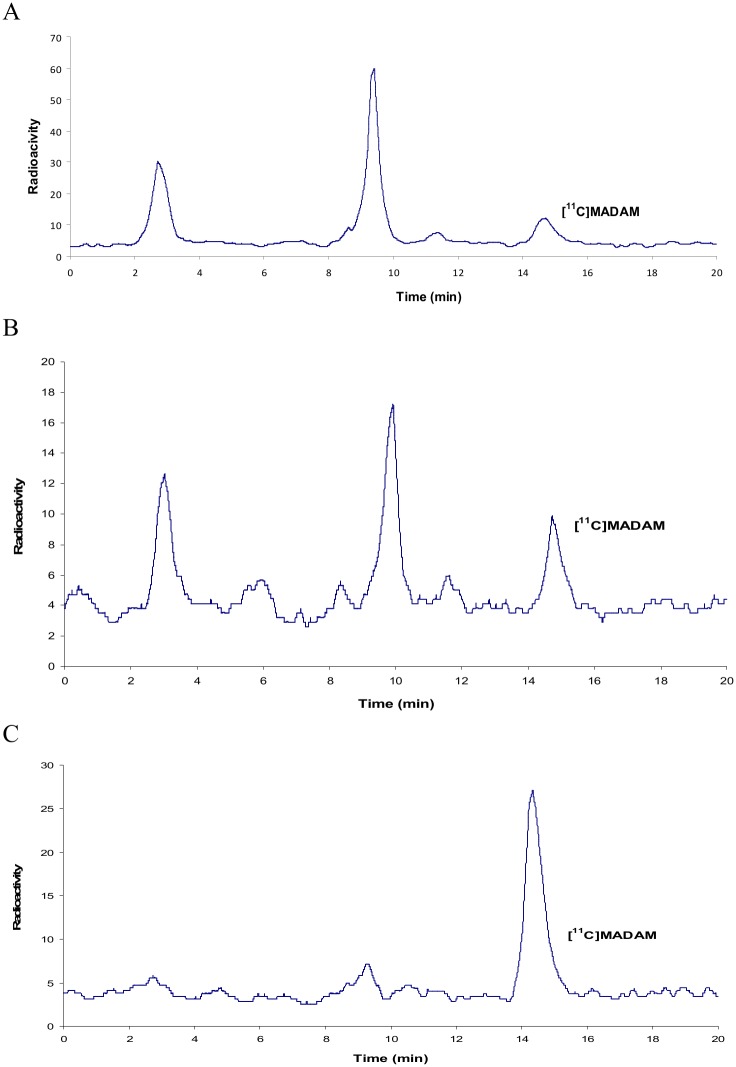
Radio-HPLC chromatograms of rat urine samples after perfusion of (A) [^11^C]MADAM, (B) [^11^C]MADAM /MADAM (25 μg) and (C) [^11^C]MADAM /MADAM (125 μg) for 15 min.

From our *in vitro* studies it was clear that the kinetics of the radiometabolism of [^11^C]MADAM were concentration-dependent and it was therefore evident that this phenomenon should be further investigated *in vivo*. In a previous study, the *in vivo* oxidation of [^11^C]MADAM into [^11^C]SOMADAM and/or [^11^C]SO_2_MADAM in rat brain was evaluated [10]. Rats were injected with [^11^C]MADAM and euthanized at 15 and 30 min post injection. Brain samples were analyzed by radio-HPLC and the chromatograms obtained showed the presence of only one radioactive peak corresponding to [^11^C]MADAM. From these results it was concluded that no radiometabolites were present in rat brain which could cross the blood-brain barrier and interfere with the interpretation and quantification of the parent tracer [^11^C]MADAM in PET studies. Since only [^11^C]MADAM was detected in rat brain, we have focused on the presence of radiometabolites in urine samples. Firstly, [^11^C]MADAM was perfused intravenously into rats over a period of time (15, 30 and 60 min). The urine samples were collected at the end of the perfusion and the radioactive compounds were analyzed by radio-HPLC. The percentage of still intact parent compound [^11^C]MADAM, as determined by radio-HPLC, was 9% after 15 min (Fig 6A) and was undetectable at 30 and 60 min. The effect of the carrier on the rate of radiometabolism of [^11^C]MADAM was investigated. Solutions containing [^11^C]MADAM and varying amounts of carrier MADAM (25 μg to 1 mg) were co-administered and urine samples were taken at the time intervals previously defined. The use of a perfusion solution of [^11^C]MADAM /MADAM (25 μg) over 15 min led to some moderate changes in the metabolism rate compared to the previous results with no carrier added [^11^C]MADAM. The percentage of unchanged [^11^C]MADAM determined by radio-HPLC was 16% (Fig 6B).

In additional *in vivo* studies using more than 25 μg carrier, no radiometabolites were observed as shown in the radiochromatogram (Fig 6C) of rat urine sample after a perfusion solution of [^11^C]MADAM /MADAM (125 μg) over 15 min in which only the parent compound [^11^C]MADAM was present. It is noteworthy that increasing the perfusion time from 30 and 60 min gave the same results, i.e. no radiometabolites were detected.

The *in vivo* results suggest that the radiometabolism of [^11^C]MADAM is dose-dependent and the results are also consistent with the data obtained in the microsomal assays. The dose-dependency of the radiometabolism to the extent seen in our study is not common. The addition of carrier to a number of tracers, such as [^11^C]PE2I and (S,S)-[^11^C]MeNER [13, 14], has been reported to not affect the radiometabolic profile of these compounds. Shetty and colleagues (2007) studied the radiometabolism of [^11^C]PE2I in rats, with a high dose of PE2I (approximately 2 mg) in order to produce sufficient amounts of metabolites for LC-MS detection without any significant changes observed in the metabolism. However in the *in vivo* experiments with [^11^C]MADAM/MADAM, no radiometabolites were detected by either radio-HPLC or by UHPLC/Q-ToF-MS when a high dose of MADAM (approximately 1 mg) was perfused into rats. Only the parent compound was present.

## Conclusions

*In vitro* metabolism by liver microsomes followed by UHPLC/Q-ToF-MS analysis is a useful approach for metabolite identification and structural elucidation. Valuable information regarding metabolism of MADAM was obtained. The rate of radiometabolism of [^11^C]MADAM was complicated, showing dose dependency both *in vitro* and *in vivo*. Even though their metabolism might be different in humans, this dose dependency for diphenyl sulfides, either as SERT radioligands or in the process of development as drug candidates, needs to be considered in PET quantifications of SERT and further investigated through more detailed studies.
